# A smart temperature and magnetic-responsive gating carbon nanotube membrane for ion and protein transportation

**DOI:** 10.1038/srep32130

**Published:** 2016-08-18

**Authors:** Hailin Cong, Xiaodan Xu, Bing Yu, Zhaohui Yang, Xiaoyan Zhang

**Affiliations:** 1 Laboratory for New Fiber Materials and Modern Textile, Growing Base for State Key Laboratory, College of Materials Science & Engineering, Qingdao University, Qingdao 266071, China; 2 College of Chemistry and Chemical Engineering, Qingdao University, Qingdao 266071, China; 3 Center for Soft Condensed Matter Physics and Interdisciplinary Research, Soochow University, Suzhou 215006, China

## Abstract

Carbon nanotube (CNT) nanoporous membranes based on pre-aligned CNTs have superior nano-transportation properties in biological science. Herein, we report a smart temperature- and temperature-magnetic-responsive CNT nanoporous membrane (CNM) by grafting thermal-sensitive poly(*N*-isopropylacrylamide) (PNIPAM) and Fe_3_O_4_ nanoparticles (Fe_3_O_4_-NPs) on the open ends of pre-aligned CNTs with a diameter around 15 nm via surface-initiated atom transfer radical polymerization (SI-ATRP) method. The inner cavity of the modified CNTs in the membrane is designed to be the only path for ion and protein transportation, and its effective diameter with a variation from ~5.7 nm to ~12.4 nm can be reversible tuned by temperature and magnetic field. The PNIPAM modified CNM (PNIPAM-CNM) and PNIPAM magnetic nanoparticles modified CNM (PNIPAM-MAG-CNM) exhibit excellent temperature- or temperature-magnetic-responsive gating property to separate proteins of different sizes. The PNIPAM-CNMs and PNIPAM-MAG-CNMs have potential applications in making artificial cells, biosensors, bioseparation and purification filters.

Biomimetics is a great interdisciplinary field which defines biologically inspired adaptation or derivation of biological functions, structures and principles of various objects from nature, design and fabrication of various similar materials as well as devices by artificial mechanisms which mimic natural ones^1^. Ion/protein channels and nuclear pore complexes (NPCs) play prerequisite roles in life, by acting as sole and effective gateway, which selectively exchanging useful ions and biomolecules between the nucleus and the cytoplasm^2^,^3^,^4^,^5^,^6^,^7^,^8^,^9^,^10^,^11^. Thus, designing a membrane contains nanochannels that mimics these complex processes in living organisms is a challenging task for scientists.

CNTs have been widely utilized for both transportation and multi-material storage through different synthetic approaches like melting-phase capillary filling or chemical vapor deposition^12^,^13^,^14^,^15^,^16^, due to its unique properties of nano-confinement effect and the interaction between the CNT and the infilling species^17^,^18^,^19^,^20^. CNM based on vertically aligned CNT array has been considered as a potential material for filtration and ion/mass transportation owing to their smooth structure and low tortuosity. This kind of membrane contains vertical aligned CNTs embedded in a crack-free matrix with open ends extending out of the film. The inner cavity of the CNTs is the only path for molecule or ion transportation^21^,^22^,^23^.

The bio-inspired nanochannels, in response to external stimulus, such as temperature, light, pH, magnetic field, and dual or even multiple stimuli, have been studied in recent years^24^,^25^,^26^. For example, a pH-responsive poly(4-vinyl pyridine) brushes were grafted on a single solid-state nanochannels by Yameen and co-workers in a fashion that led to a switchable electronic readout of nanodevices^27^. Wen *et al*. demonstrated an artificial functional nanopore based on polyimide as an ionic gate whose transport properties could be changed by UV irradiation and pH, exhibiting ionic current rectifying behavior^28^,^29^. Additionally, there exist some bio-inspired nanostructures sensing biological molecules. Chen *et al*. developed a silicon microcolumn array-based substrate with regular arranged conical spikes at different processing environment^30^. The radii of the spike tips ranged from 500 nm in water, 2 μm in SF_6_ gas and 5 μm in air. Thus, peptide mass spectra directly induced confirmed the formation of protonated ions of different peptides from the processed silicon surface. Shao *et al*. fabricated wire arrays with deposited gold nanoparticles for electric detection of DNA molecules^31^. A sensitive detection of DNA molecules as low as 1 pM was achieved because of the high surface to volume ratio of the porous structures. They also detected the interaction between DNAs and proteins. Such membranes containing smart nanochannels could potentially spark theoretical and experimental efforts to simulate the process of ion and protein transport in living systems and boost the blossom of biomimic membranes.

PNIPAM is a typical thermal-responsive biocompatible polymer with a lower critical solution temperature (LCST) of 32 °C in water. It has been widely used in the fabrication of smart gating membranes^32^,^33^,^34^,^35^,^36^,^37^. PNIPAM chains are stretched when the temperature below LCST due to the formation of hydrogen bonds with water, resulting in “close” state of the membrane. When the temperature is higher than LCST, PNIPAM chains are shrunken due to the broken of hydrogen bonds with water, resulting in “open” state of the membrane^38^,^39^,^40^.

Inspired by the above results, we prepared a new temperature- and temperature-magnetic-responsive CNM based on vertically pre-aligned CNT arrays by grafting temperature sensitive poly(N-isopropylacrylamide) (PNIPAM) and magnetic Fe_3_O_4_-NPs on the open ends of CNTs. Magnetic Fe_3_O_4_-NPs had been used in membrane fields^41^,^42^. The polymer chains connected with Fe_3_O_4_-NPs are stretched vertically to the membrane surface when the magnetic field is located in the direction which is perpendicular to the film surface, resulting in “open” state of the membrane. When the magnetic field is removed from the membrane, polymer chains connected with Fe_3_O_4_-NPs are aggregated on the membrane surface, resulting in “close” state of the membrane. As illustrated in Fig. 1a, the CNT array was grown vertically on the substrate by chemical vapor deposition (CVD) method and the CNT/epoxy nanoporous membranes with a thickness of around 10 μm were prepared through epoxy embedment and microtome cutting procedures. The prepared CNT/epoxy nanoporous membranes were processed through a six-step functionalization procedure to graft PNIPAM on the open ends of CNTs (Fig. 1b). Firstly, the open ends of CNTs were oxidized and hydrolyzed to form carboxyl groups. Then, the bromine-terminated monomers attached on the open ends of CNTs were prepared by a series of esterification and bromination reactions. PNIPAM chains were grafted on the surface of membrane through surface-initiated atom transfer radical polymerization (SI-ATRP) method. The end containing amine group and Fe_3_O_4_-NPs was generated by bromide group through Gabriel synthesis. The fabrication and modification of CNM as well as the ion and protein transportation properties of the obtained PNIPAM-CNM and PNIPAM-MAG-CNM were studied and discussed in this paper.

## Results and Discussion

**Figure 2:**
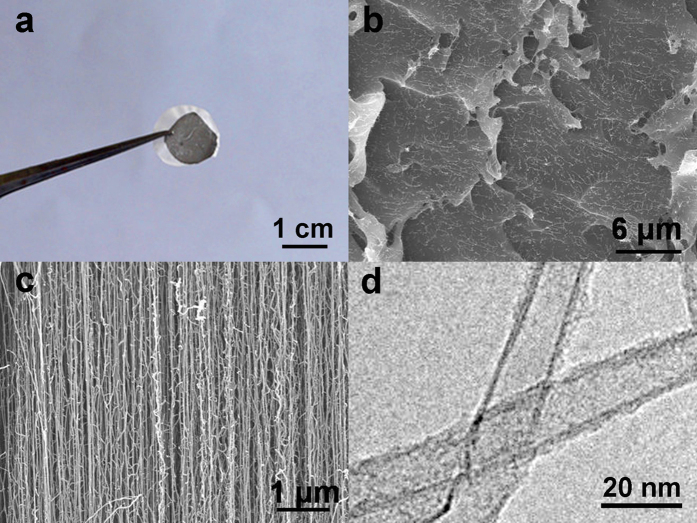
Images of the obtained CNM: (**a**) optical photo, (**b**) top surface of membrane by SEM, (**c**) cross section surface of membrane by SEM, (**d**) TEM image of CNTs in membrane.

Figure 2a shows the photo of the sliced CNM after being embedded in epoxy resin and microtome cutting. From the top view of pure CNM, many CNT ends extend out of the membrane surface (Fig. 2b). The cross sectional image of the membrane is shown in Fig. 2c, indicating that the CNTs in the epoxy retain vertically aligned formation very well. Figure 2d shows the TEM image of the CNTs in the CNM with an average diameter of ~15 nm.

**Figure 3:**
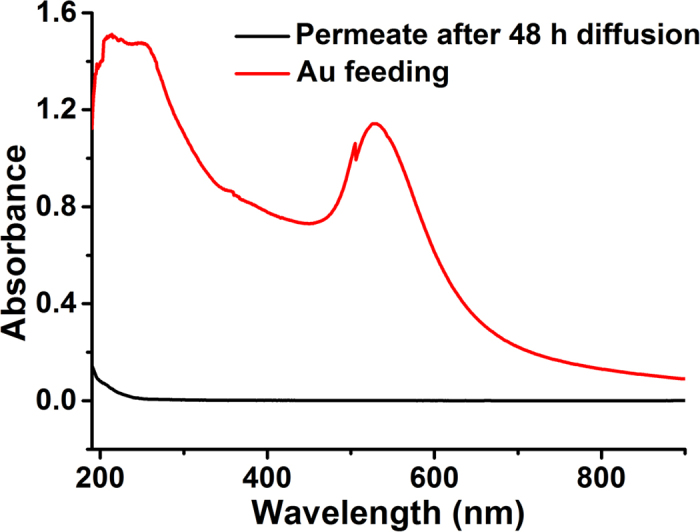
UV-Vis spectra of Au-NPs feed suspension (red curve) and the permeate solution (black curve) after diffusion for 48 hours in the obtained CNM.

In order to verify that the pore size of the CNM is <16 nm and no macrocracks exist inside the CNM, we use monodisperse gold nanoparticles (Au-NPs) with diameter of 16 nm to do the size exclusion test^43^. Compared the ultraviolet-visible (UV-Vis) spectra of gold nanoparticle feed suspension (red curve) and the permeate solution (black curve) after diffusion for 48 hours in the CNM (Fig. 3), there is no obvious absorption peak of Au-NPs in the permeate solution, which demonstrates that pore size of the CNM is smaller than 16 nm and no macrocracks are present in the CNM.

**Figure 4:**
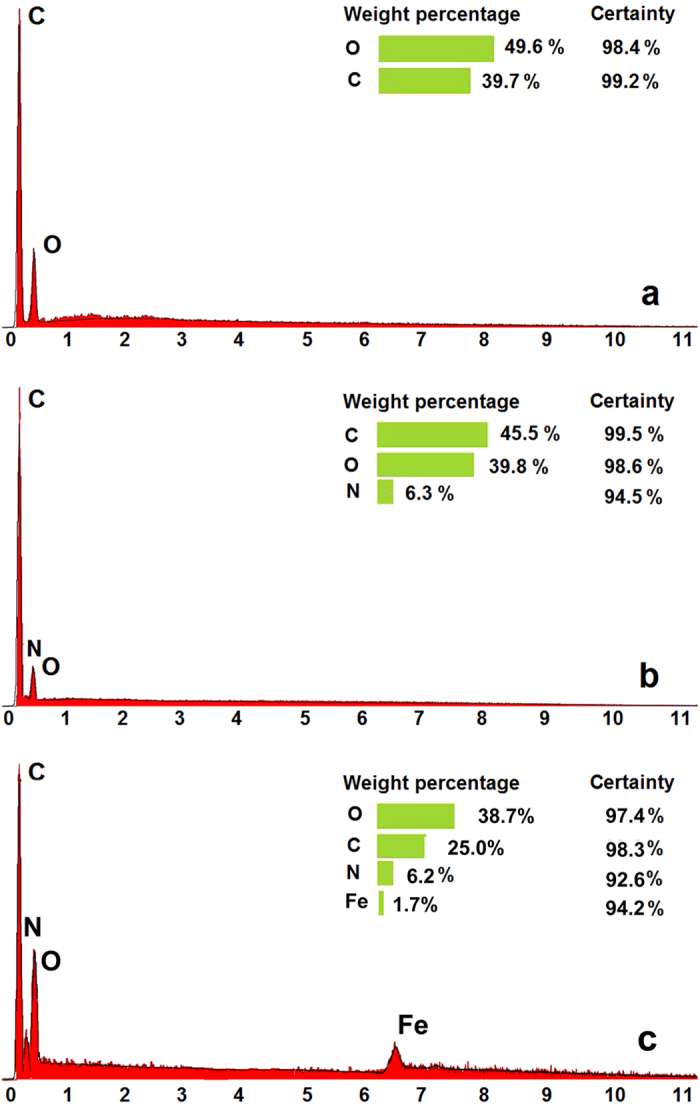
EDS spectra of pure CNM (**a**), PNIPAM-CNM (**b**) and PNIPAM-MAG-CNM (**c**).

After surface modification, the energy dispersive spectroscopy (EDS) tests are operated to confirm that PNIPAM and Fe_3_O_4_-NPs are successfully grafted on the surfaces of CNM. Figure 4a,b,c show the EDS spectra of the pure CNMs, PNIPAM-CNMs and PNIPAM-MAG-CNMs, respectively. The pure CNMs contain ~49.6% of element O and ~39.7% of element C, while the PNIPAM-CNMs contain ~39.8% of element O, ~45.5% of element C, and ~6.3% of element N. The existence of element N in PNIPAM-CNM sample indicates the temperature-responsive PNIPAM is successfully grafted on the CNMs. At the meantime, the PNIPAM-MAG-CNMs contain ~38.7% of element O, ~25.0% of element C, ~6.2% of element N, and ~1.7% of element Fe, which indicate PNIPAM and magnetic particles are successfully modified on the pure CNMs. In our study, the calculated grafting yield of PNIPAM is 0.0701–0.1203 mg cm^−2^ by weight increase of the CNM after modification with different SI-ATRP reaction time. The 30 minutes reaction time in the middle is chosen for the following experiments, which was decided by the results from Supplementary Table S1.

**Figure 5:**
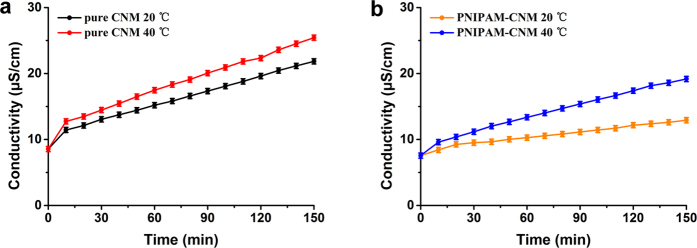
Ion transportation properties of (**a**) the pure CNM at 20 °C and 40 °C, and (**b**) PNIPAM-CNM at 20 °C and 40 °C measured through conductivity variation in the downstream.

The ion transportation properties and effective pore sizes of the pure CNM and PNIPAM-CNM can be measured by salt diffusion test, because the trans-film permeability of ions can be measured by conductivity change in downstream of membrane and can be correlated to the effective pore sizes by Knudsen diffusion law^17^. As the most common substance contains cations and anions, KCl aqueous solution is chosen to detect the ion transportation of membranes^14^,^18^,^22^. Figure 5 shows the conductivity change in downstream of the pure CNM (a) and PNIPAM-CNM (b) at different temperatures with different penetration times. For two kinds of CNMs, the downstream conductivity increases with the extension of penetration time. However, the downstream conductivity of the PNIPAM-CNM is lower than that of pure CNM at the same conditions. It can be explained that the effective pore size decreases due to the introduction of PNIPAM polymer. After diffusion for 150 min, we can see that the conductivity variation of PNIPAM-CNM is 2.21 times larger than that of pure CNM when temperature rising from 20 to 40 °C, because the effective pore size of the PNIPAM-CNM changes along with the conformation variation of PNIPAM polymer chains at different temperatures.

**Figure 6:**
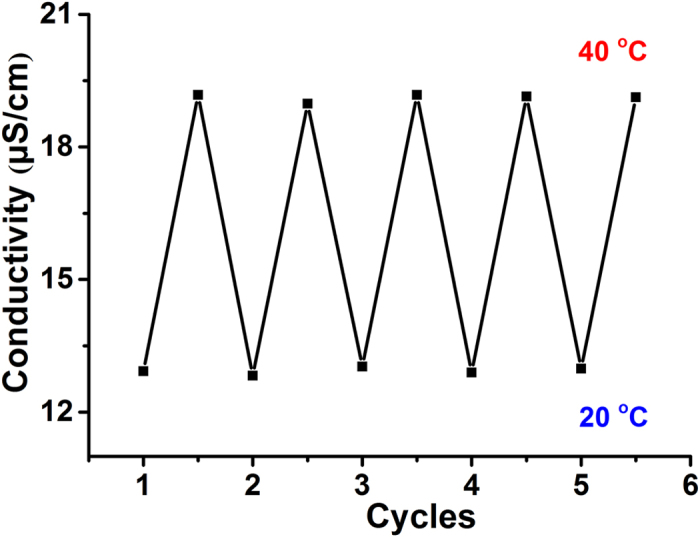
Reversible temperature-responsive property of the PNIPAM-CNM for ion transportation measured through conductivity variation in the downstream.

In order to know the repeatability of the PNIPAM-CNM for ion transportation at different temperatures, we test the variation of the downstream conductivity after 150 min for 5 cycles at 20 and 40 °C. As presented in Fig. 6, the conductivity values are very similar at different temperature after 5 cycles, indicating the reversible temperature-responsive property of the smart PNIPAM-CNM. Therefore, the thermo-sensitive PNIPAM-CNM can be used as gating membrane to achieve an accurate and reversible control of ion/molecule transportation.

The trans-film permeability can be correlated to the effective pore sizes by Knudsen diffusion law, as shown in equation (1)^17^:


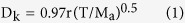


where, D_k_ stands for Knudsen diffusion coefficient which is proportional to the downstream conductivity change over time, T is the absolute temperature, M_a_ is the molecular weight of the permeate molecule, and r represents the pore radius. The radius ratio of pores after and before modification could be estimated via equation (2):


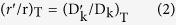


where, r′ stands for the effective pore radius of PNIPAM-CNM, r represents the effective pore radius of pure CNM. The pore diameter of pure CNM is ~15 nm at 20 °C. Because of the low coefficient of thermal expansion of CNTs, the pore diameter of pure CNM at 40 °C can also be considered as ~15 nm. We assume that the downstream conductivity variation over time was an approximately linear relationship. At the first ten minutes, unsteady ion diffusion process with considerable impulse is generated during the injection of high concentration KCl solution into one side of the testing cells. Thus, the average slope can be calculated according to the conductivity variation over time between 10 to 150 minutes. The average slopes of pure CNM and PNIPAM-CNM at different temperatures are shown in Supplementary Table S1. The average slopes of pure CNM at 20 and 40 °C are 0.0743 and 0.0906, respectively. Similarly, the slopes of PNIPAM-CNM at 20 and 40 °C are 0.0322 and 0.0683 from Fig. 5b, respectively. Thus, (r′⁄r) at 20 °C is calculated to be 0.4334. Similarly, the calculated value of (r′⁄r) at 40 °C is about 0.7538. Therefore, the effective pore diameters of PNIPAM-CNM in 20 °C and 40 °C are calculated to be 6.50 nm and 11.31 nm, respectively. These results clearly evidence the nanovalving behavior of the grafted PNIPAM in the PNIPAM-CNMs. The thermo-responsive PNIPAM acts as a thermally driven gate controlling the ionic flow through the PNIPAM-CNM. As can be seen from Supplementary Table S2, the degree of grafting is well controlled with reaction time which shows the great advantage of SI-ATRP method. Through the above data, we can calculate the effective nanochannel diameters at 20 °C of different SI-ATRP reaction time which are also shown in Supplementary Table S2.

Because unsteady ion diffusion process with considerable impulse is generated during the injection of high concentration KCl solution into one side of the testing cells at the first ten minutes, it might bring uncertainty to the radius calculation. Therefore, an error calculation is curried out. The calculated diameters of PNIPAM-CNM without or with the first ten minutes data are listed in Supplementary Table S3, and the result shows that the calculated errors are relatively small, which means the data in unsteady diffusion process have a very little influence on the calculated diameters.

**Figure 7:**
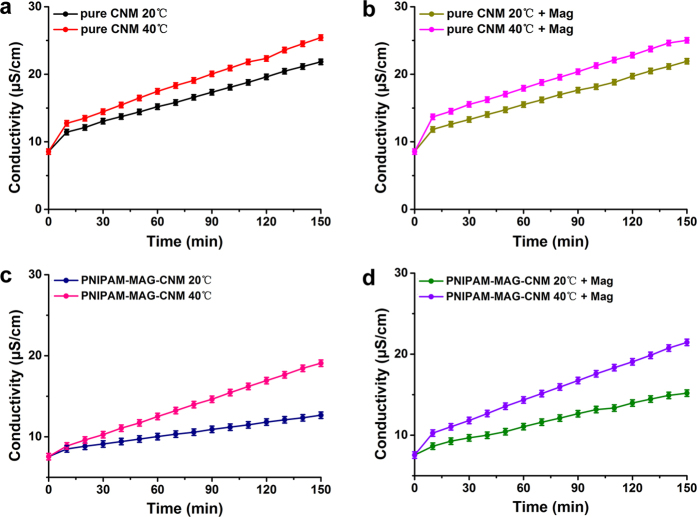
Ion transportation properties of the (**a**) pure CNM at 20 °C and 40 °C without magnetic field, (**b**) pure CNM at 20 °C and 40 °C with magnetic field, (**c**) PNIPAM-MAG-CNM at 20 °C and 40 °C without magnetic field, and (**d**) PNIPAM-MAG-CNM at 20 °C and 40 °C with magnetic field measured through conductivity variation in the downstream.

Similarly, the ion transportation properties of the pure CNM and PNIPAM-MAG-CNM can be measured by KCl diffusion test under different temperatures and magnetic field. The magnetic field (0.3 T) was placed in a vertical direction of the membrane surface. Figure 7 shows the conductivity change in downstream of the pure CNM (a and b) and PNIPAM-MAG-CNM (c and d) at different temperatures and magnetic field with different penetration times. For two kinds of CNMs, the downstream conductivity increases with the extension of penetration time. However, the downstream conductivity of the PNIPAM-MAG-CNM is lower than that of pure CNM and PNIPAM-CNM (Fig. 5) at the same conditions. It can be explained that the effective pore size decreases due to the introduction of PNIPAM polymer and magnetic nanoparticles. After diffusion for 150 min, we can see that the conductivity variation of PNIPAM-MAG-CNM is much larger than that of pure CNM when temperature rising from 20 to 40 °C, because the effective pore size of the PNIPAM-CNM changes along with the conformation variation of PNIPAM polymer chains at different temperatures. And the conductivity variation of PNIPAM-MAG-CNM is also larger than that of pure CNM when magnetic field changing from none to existence, because the effective pore size of the PNIPAM-MAG-CNM changes along with the conformation variation of Fe_3_O_4_-NPs chains at different magnetic environments. As shown in Fig. 7a,b, there is no significant change of conductivity for pure CNM under or not under the magnetic environment. The average slopes of PNIPAM-MAG-CNM at different temperatures and magnetic field are shown in Supplementary Table S4. Similarly, the effective pore diameters of PNIPAM-MAG-CNM in 20 °C and 40 °C without outer magnetic field are calculated based on equation 2 to be 6.00 nm and 12.08 nm, respectively. And the effective pore diameters of PNIPAM-MAG-CNM in 20 °C and 40 °C with outer magnetic field are calculated to be 9.43 nm and 13.23 nm, respectively. These results clearly evidence the nanovalving behavior of the grafted PNIPAM and magnetism of Fe_3_O_4_-NPs in the PNIPAM-MAG-CNMs.

**Figure 8:**
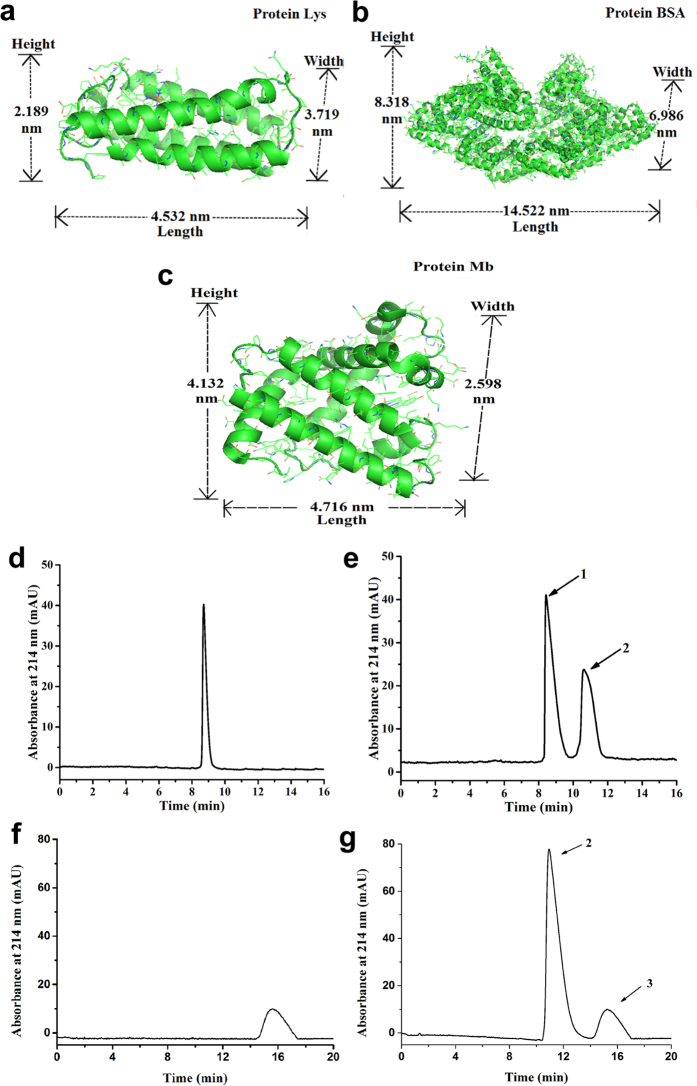
Dimension figures of Lys (**a**), BSA (**b**), Mb (**c**), and CE detection of filtrated proteins by PNIPAM-CNM at (**d**) T = 20 °C, (**e**) T = 40 °C, and by PNIPAM-MAG-CNM at (**f**) T = 20 °C without magnetic field, (**g**) T = 20 °C with magnetic field. Separation conditions: buffer, 40 mM phosphate (pH = 3.0); injection, 20 s with a height difference of 20 cm; applied voltage, +15 kV; UV detection, 214 nm; sample, 0.5 mg·mL^−1^ for each protein; capillary, 75 μm id × 50 cm (40 cm effective); capillary temperature, 25 °C. Peak identification: (1) Lys; (2) BSA; (3) Mb.

Because the PNIPAM-CNM shows thermo-controllable nanopore diameters at different temperatures and magnetic field, it can be used as a nanofiltration membrane to separate proteins of different sizes. Figure 8d,e show capillary electrophoresis separation results for mixed proteins which are filtrated through the temperature-responsive PNIPAM-CNM at 20 °C and 40 °C, respectively. The two proteins are lysozyme (Lys), which has a molecular width of about 3.719 nm (Fig. 8a), and bovine serum albumin (BSA), which has a molecular width of about 6.986 nm (Fig. 8b), respectively. The PNIPAM-CNM has smaller pore size at 20 °C, which could be used for selectively transportation of Lys (Fig. 8d). The pore size of PNIPAM-CNM becomes larger at 40 °C, thus BSA and Lys can be both transported (Fig. 8e). Moreover, when the protein is exposed *in vitro* environment, its functional groups and structure are prone to lose its original activity and become unstable especially when the temperature changes. The natural folded structure of protein becomes a completely unfolded structure at a certain temperature which is called denature temperature. The apparent denature temperatures of BSA, Mb and Lys are 70, 72 and 187 °C, respectively^44^,^45^,^46^. Thus, proteins used in our experiments keep their nature structures under 20 and 40 °C. Figure 8f,g show capillary electrophoresis separation results for mixed proteins which are filtrated through the PNIPAM-MAG-CNM at 20 °C without and with magnetic field, respectively. The magnetic field (0.3 T) was placed in a vertical direction of the membrane surface. The two proteins are bovine serum albumin (BSA), which has a molecular width of about 6.986 nm (Fig. 8b), and myoglobin (Mb), which has a molecular width of about 2.598 nm (Fig. 8a), respectively. The PNIPAM-MAG-CNM has smaller pore size at 20 °C without magnetic field, which could be used for selectively transportation of Mb (Fig. 8f). The pore size of PNIPAM-CNM becomes larger under magnetic environment, thus BSA and Mb can be both transported (Fig. 8g). Therefore, the smart CNMs have potential applications in making artificial cells, biosensors, bioseparation and purification filters^47^.

## Methods

### Materials

Ethylene (C_2_H_4_, 99.999%), hydrogen gas (H_2_, 99.999%), and argon gas (Ar, 99.999%) were bought from Chengdu Keyuan Gas Company. SPI-pon-812 monomer, DDSA, nmA and DMP-30 as the precursor were supplied by SPI Supplies. Potassium chloride (KCl, 99.5%), potassium permanganate (KMnO_4_, 99.5%), sulfuric acid (H_2_SO_4_, 98.0%) and hydrochloric acid (HCl, 37.5%) were bought from Tianjin Hengxing Chemical Reagent Company. Methanol (99.9%), and ethanol (99.9%) were obtained from Tianjin Fuyu Chemical Company. N,N’-diisopropylcarbodiimide (DPCI, 98%), 1-hydroxy-benzotriazolehydrate (HOBth, 97.0%), dimethylformamide (DMF, 99.9%), ethanolamine (99.0%), triethylamine (99.5%), 4-N,N-dimethylaminopyridine (DMAP, 99.0%), α-bromoisobutyrlbromide (98.0%), anhydrous acetonitrile (99.8%), N-isopropylacrylamide (NIPAM, 98%), 2,2’-bipyridine (BPy, 99.0%), cuprous chloride (CuCl, 97.0%), copper (II) chloride (CuCl_2_, 98%), cupric bromide (CuBr_2_, 99.0%), N,N,N’,N”,N”-pentamethyldiethylenetriamine (PMDETA, 99.0%), potassium phthalimide salt (98.0%), hydrazine hydrate (98.0%), 1-ethyl-3-(3-dimethylaminopropyl) carbodiimide (EDC, 98.0%) and N-hydroxysuccinimide (NHS, 98.0%) were bought from Aladdin Industrial Corporation. Chloroauric acid (HAuCl_4_, Au: 48–50%) was bought from Sinopharm Chemical Reagent Company. Sodium citrate (Na_3_C_6_H_5_O_7_, 99.0%) was brought from Shanghai Aibi Chemical Reagent Company. All the chemicals were used as received until noted elsewhere. Monodisperse gold suspension with particle diameter of 16 nm was synthesized using a procedure reported elsewhere^43^.

### Membrane preparation

We used the modified CVD method to grow vertically aligned multi-walled CNT arrays in a horizontal quartz tube furnace with a one-inch diameter quartz tube (Winston-salem NC Instruments) at 750 °C. The Si wafer was pre-coated with a 100 nm SiO_2_ layer and an Al/Fe catalyst layer. Ethylene (120 sccm) was used as the carbon source. H_2_ (100 sccm) and Ar (160 sccm) were used as a co-gas source. The CNT arrays with thickness of 100–500 μm could be fabricated after 5–10 min growth. The CNT arrays obtained above were then mixed with an epoxy precursor solution and cured in a vacuum oven. After curing at 80 °C overnight and then cutting by using a microtome (RMC, Boeckeler Instruments), the sliced open-end CNMs with thickness of 10 μm were prepared successfully.

### Membrane modification

The membrane modification was performed in the following four-step functionalization procedures. The first step was oxidation and hydrolysis of CNM. The oxidative solution was prepared from 1.25 g of KMnO_4_ in 25 mL of H_2_SO_4_ (0.375 mol·L^−1^). The CNM sample was placed in small tube and 3 mL of above mentioned solution was added to the tube. After 30 min with gentle shaking, the CNM was removed and washed with de-ionized water, HCl (6 mol·L^−1^), deionized water and ethanol, respectively. Then, the acidulated CNM was dried in a vacuum oven at 45 °C for 30 min. The second step was pre-functionalization of above sample. The pre-functionalized solution was prepared from 0.3833 g of HOBth and 0.1583 g of DPCI in 25 mL of DMF. The CNM was placed in small tube with 3 mL of above mentioned solution for about 30 min under gentle shaking. After being washed with DMF twice, the CNM was reacted with 0.7633 g of ethanolamine immediately in 25 mL of DMF for 2 h under gentle shaking. Then, the CNM was removed and washed with DMF and ethanol, dried in a vacuum oven at 45 °C for 30 min. The third step was initiator immobilization of CNM. The initiated solution was prepared from 0.2525 g of triethylamine, 0.01525 g of DMAP, and 0.46 g of α-bromoisobutyrlbromide in 25 mL of anhydrous acetonitrile. The CNM was reacted in the above mentioned solution for 2 h under the room temperature. The obtained CNM was used directly in the next step without further treatment. The last step which was the most important was SI-ATRP of CNM. The CNM was added to a mix solution of NIPAM, CuCl, CuCl_2_ and BPy in water and methanol (v/v = 1 : 1) with molar ratios of 100 : 0.5 : 0.1 : 1.5 for 20–50 min at room temperature. Then the membrane was placed in a quenching solution containing 500 mg of CuBr_2_ and 1.25 mL of PMDETA in 100 mL of water and methanol (v/v = 1 : 1) for 30 min at room temperature to stop the whole polymerization. After that, the membrane was removed and washed with de-ionized water, methanol and ethanol for three times. Finally, the obtained PNIPAM-CNM was dried in a vacuum oven at 45 °C for 12 hours. The PNIPAM-CNM could be further modified to PNIPAM-MAG-CNM through Gabriel synthesis and nanoparticles coupling. Firstly, the PNIPAM-CNM was reacted in a 60 °C oil bath for 6 h in a mixture of 100 g DMF and 3 g potassium phthalimide salt with gentle shaking. Then the membrane was reacted in a solution of 7 mL hydrazine hydrate in 25 mL of 6 M HCl for 6 h at 60 °C with gentle shaking. Finally, the membrane above was reacted with a solution of 31.2 mg EDC, 38.7 mg NHS, 0.3 mL nanoparticle solution and 10 mL water for 4 h in the dark. After the reaction, the PNIPAM-MAG-CNM was removed and washed with de-ionized water, methanol and ethanol for three times. Finally, the obtained PNIPAM-MAG-CNM was dried in a vacuum oven at 45 °C for 12 hours. The oleic acid modified Fe_3_O_4_-NPs with ~7 nm were fabricated using a procedure reported elsewhere^48^.

### Instruments

SEM images were taken on a Hitachi S-4700 microscope. TEM images were obtained on a Tecnai G220 (FEI) microscope at 180 kV accelerating voltage. UV-Vis spectra were operated on the TU-1810 UV-visible spectrophotometer (Beijing Puxi General Instrument Company). For the permeability test, a pure CNM or PNIAPM-CNM was mounted between two chambers of the conductivity cells as shown in Supplementary Figure S1a. One half of the chamber was filled with 10^−4^ mol·L^−1^ of KCl, another one was filled with 10^−6^ mol·L^−1^ of KCl. The conductivity change of KCl with lower downstream concentration was recorded by a DDS-11A conductivity meter (Shanghai Shengci Instrument Company). At the meantime, the protein filtration was carried out by the device as shown in Supplementary Figure S1b. The CNM was placed in the middle of a permeation module which connected with mixed protein solution. The filtrated proteins were detected by a CL1020 capillary electrophoresis instrument (Huayang liming instrument Company). Fused-silica capillary of 75 μm i.d. and 375 μm o.d. was provided by Hebei Yongnian Optic Fiber Company.

## Conclusions

In conclusion, temperature- and temperature-magnetic-responsive carbon nanotube membranes were fabricated successfully by CVD and SI-ATRP methods. The PNIPAM polymers grafted on the CNMs served as temperature-responsive nanovalves on the open ends of pre-aligned CNTs. The Fe_3_O_4_-NPs modified on the CNMs served as magnetic-responsive nanovalves. The permeability of the CNMs in response to temperature and magnetic field were investigated by measuring the downstream conductivity caused by ion diffusion of KCl aqueous solutions. PNIPAM chains were stretched when the temperature below LCST, resulting in “close” state of the nanopores in the membrane. When the temperature was higher than LCST, PNIPAM chains were shrunken, resulting in “open” state of the nanopores in the membrane. At the meantime, the chains grafted with Fe_3_O_4_-NPs swung in magnetic field direction. Proteins of different sizes were successfully separated through the PNIPAM-CNMs by tuning temperature and through the PNIPAM-MAG-CNM by changing magnetic field. The smart thermo-sensitive PNIPAM-CNMs and thermo-magnetic-sensitive PNIPAM-MAG-CNMs showed potential applications in making artificial cells, biosensors, bioseparation and purification filters.

## Additional Information

**How to cite this article**: Cong, H. *et al.* A smart temperature and magnetic-responsive gating carbon nanotube membrane for ion and protein transportation. *Sci. Rep.* **6**, 32130; doi: 10.1038/srep32130 (2016).

## Supplementary Material

Supplementary Information

## Figures and Tables

**Figure 1:**
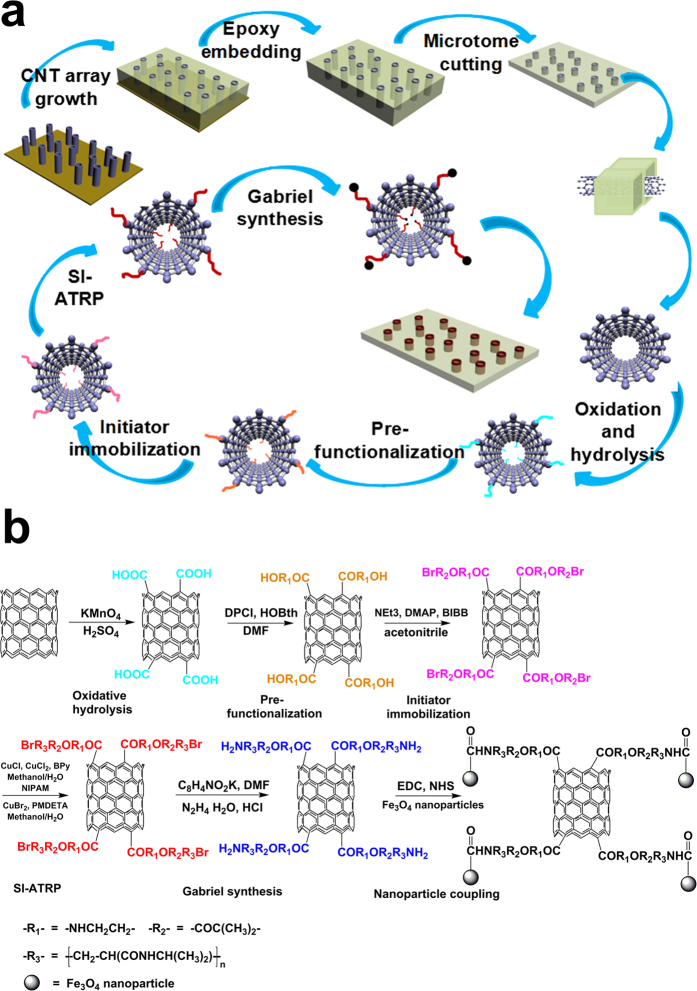
Schematic (**a**) and chemical (**b**) illustration of the fabrication process of PNIPAM-MAG-CNM.
