# Supplementary material for: A smart temperature and magnetic-responsive gating carbon nanotube membrane for ion and protein transportation
Source: Sci Rep. 2016 Aug 18;6:32130. doi: 10.1038/srep32130 (PMC4989442; doi:10.1038/srep32130)
Supplement: Supplementary Information [file srep32130-s1.doc]

A smart temperature and magnetic-responsive gating carbon nanotube membrane for ion and protein transportation

Hailin Cong*, Xiaodan Xu, Bing Yu*, Zhaohui Yang*, Xiaoyan Zhang

^1^Laboratory for New Fiber Materials and Modern Textile, Growing Base for State Key Laboratory, College of Materials Science & Engineering, Qingdao University, Qingdao 266071, China.

^2^College of Chemistry and Chemical Engineering, Qingdao University, Qingdao 266071, China.

^3^Center for Soft Condensed Matter Physics and Interdisciplinary Research, Soochow University, Suzhou 215006, China.

^*^hailincong@yahoo.com; yubingqdu@yahoo.com.

**Table S1.** Slopes of KCl diffusion test curves of pure CNM and PNIPAM-CNM at different temperatures

| Membranes | 20 °C | 40 °C |
| --- | --- | --- |
| Pure CNM | 0.0743 ± 0.0057 | 0.0906 ± 0.0058 |
| PNIPAM-CNM | 0.0322 ± 0.0057 | 0.0683 ± 0.0058 |

**Table S2.** Degree of grafting and effective nanochannel diameters with different SI-ATRP reaction time for PNIPAM-CNM

| SI-ATRP reaction time (min) | 20 | 30 | 40 | 50 |
| --- | --- | --- | --- | --- |
| Degree of grafting (mg·cm^-2^) | 0.0701 | 0.0892 | 0.1097 | 0.1203 |
| Nanochannel diameter (nm) | 8.32 | 6.50 | 4.55 | 3.54 |

**Table S3.** Calculated nanochannel diameters of PNIPAM-CNM under steady and unsteady diffusion process at different temperatures

| Nanochannel diameter (nm) | Steady diffusion | Unsteady diffusion | Error calculation |
| --- | --- | --- | --- |
| PNIPAM-CNM (20 °C) | 6.50 | 6.08 | 6.46% |
| PNIPAM-CNM (40 °C) | 11.31 | 10.32 | 8.75% |

**Table S4.** Slopes of KCl diffusion test curves of PNIPAM-MAG-CNM at different temperatures and magnetic fields

| Membranes | 20 °C with magnetic fields | 40 °C with magnetic fields | 20 °C without magnetic fields | 40 °C without magnetic fields |
| --- | --- | --- | --- | --- |
| PNIPAM-MAG-CNM | 0.0297 ± 0.0057 | 0.0730 ± 0.0057 | 0.0467 ± 0.0057 | 0.0799 ± 0.0057 |


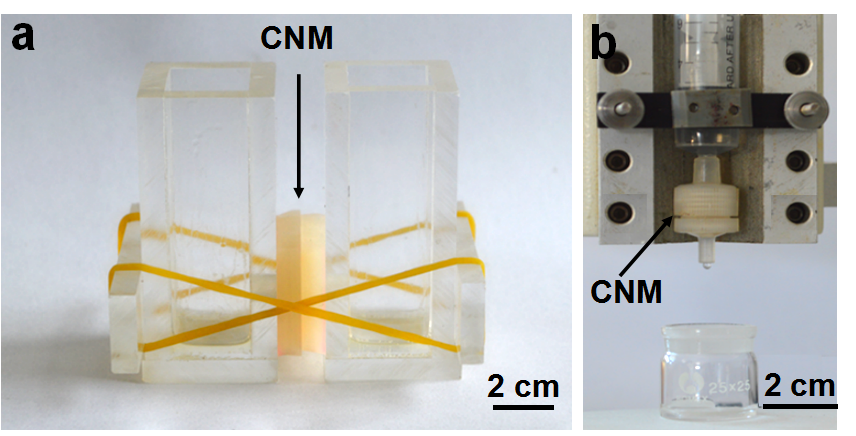


**Figure S1.** The digital pictures of the conductivity cells for measuring conductivity (a) and the filtering device for proteins (b).
